# The Intricate Nonadiabatic Dynamics of NO^+^ and NO_3_^–^ Mutual Neutralization

**DOI:** 10.1021/jacs.6c03637

**Published:** 2026-05-25

**Authors:** Alon Bogot, Mathias Poline, Ming Chao Ji, Arnaud Dochain, Paul Martini, Stefan Rosén, Henning Zettergren, Henning T. Schmidt, Richard D. Thomas, Daniel Strasser

**Affiliations:** † Institute of Chemistry, 26742 The Hebrew University of Jerusalem, Jerusalem 9190401, Israel; ‡ Department of Physics, 7675 Stockholm University, Stockholm SE-10691, Sweden; § Institute of Condensed Matter and Nanosciences, Universite Catholique de Louvain, Louvain-la-Neuve B-1348, Belgium

## Abstract

Mutual neutralization reactions play a subtle but crucial role in atmospheric chemistry. The intrinsic gap between typical ionization energies and electron affinities allows cation–anion reactions to produce a broad range of neutral products. Specifically, mutual neutralization of NO^+^ and NO_3_^–^ ions in the presence of water has been proposed to play a key role in the formation of atmospheric nitrous acid (HONO) and, consequently, also OH radical formation. Nevertheless, the mechanisms and products of molecular anion–cation reactions are largely unknown. Here, we present a detailed experimental study of the isolated NO^+^ and NO_3_^–^ reactions, using three-dimensional coincidence imaging of the neutral products of low-energy collisions in merged cation and anion beams. We found that while 15 product channels are energetically accessible, the reaction proceeds exclusively via a single nonadiabatic pathway yielding NO + NO_2_ + O. Momentum correlation analysis revealed an intricate nonadiabatic mechanism, initiated by a long-range electron transfer at ∼6 Å distance between the ions, resulting in vibrationally hot NO and an electronically excited NO_3_ (^2^E′) intermediate that undergoes subsequent dissociation, attributed to a conical intersection with the lower lying NO_3_ (^2^E″) state. The mechanistic picture of NO^+^ + NO_3_^–^ neutralization and identification of specific intermediates provides a basis for considering the competing processes that in the presence of water can lead to HONO formation.

## Graphical abstract

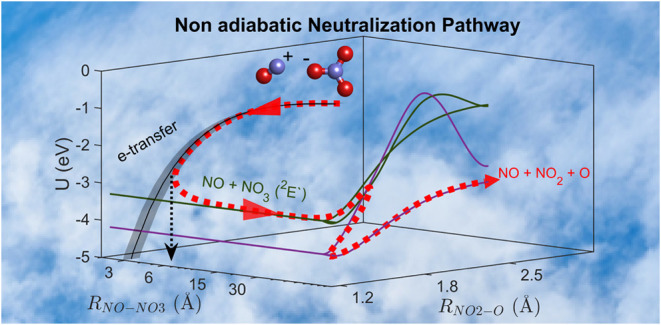

## Introduction

Molecular ion neutralization processes are important in driving chemical evolution in many partly ionized gas-phase environments such as the interstellar medium,^1–3^ planetary atmospheres,^4,5^ as well as man-made plasma in semiconductor and tokomak applications.^6–9^ In liquid environments, ion neutralization mechanisms have a well-known role in acid–base chemistry.^10,11^ Recent studies suggested that less-known ion neutralization mechanisms occurring at the liquid–air interface can be responsible for several surprising experimental observations, including the spontaneous formation of OH radicals and hydrogen peroxide on water microdroplets,^12–15^ which is a subject of controversy,^16–21^ as well as the daytime formation of atmospheric nitrous acid (HONO) concentrations that are not explained by known gas-phase photochemical reactions.^22–24^

The merged ion-beams technique makes it possible to study isolated mutual neutralization (MN) reactions of cations and anions in the relevant internal excitation and collision energy conditions.^25–27^ Combined with ion-beam trapping,^28,29^ it is possible to study MN reactions of molecular ion systems that are produced in a hot state but can undergo thermalization to the ambient temperature of the ion beam storage device.^28–34^ Recent studies, performing 3D imaging of the neutral products of isolated reactions of hydronium cations and hydroxide anions, provided detailed insight into the competing nonadiabatic pathways for OH radical formation in the MN process.^35,36^ In contrast to hydronium and hydroxide neutralization in bulk water that is expected to be dominated by proton-transfer and form two water molecules, neutralization of isolated water ions is dominated by electron-transfer (ET) dynamics and leads to efficient OH formation. Such efficient formation of OH radicals in MN reactions of partly solvated ions at a water–air interface may account for hydrogen peroxide on water microdroplets.^12–21^

Atmospheric HONO, which is another source for OH radicals,^37,38^ is proposed to form via heterogeneous hydrolysis and charge separation at the contact of a pair of NO_2_ molecules with the liquid water surface.^39–45^ Here, the MN of the resulting NO^+^ and NO_3_^–^ ions in the presence of water was proposed to release HONO back to the gas phase.^39–44^ It is therefore interesting to explore isolated MN reactions of NO^+^ and NO_3_^–^ ions and experimentally probe the resulting neutral products and the underlying mechanism. In the absence of water, we expect ET to be the dominant MN mechanism, yielding neutral NO and NO_3_ radicals, which may react further. Early flowing afterglow experiments studied the NO^+^ + NO_3_^–^ MN rate, albeit, using hot ions directly from a microwave discharge or electron-bombardment ion sources with undetermined and broad internal energy distributions.^46–49^ Ion trapping allows for exploring the reactions of internally cold ions, relieving the initial state uncertainty.^28–34^ Nevertheless, even when the reacting ions are vibrationally cold, the large ∼5.3 eV gap between the NO ionization potential^50^ and NO_3_ electron affinity^51^ provides abundant excess energy that can lead to rich dynamics in the neutral product channels. Electronically excited NO (^4^Π) can be energetically accessible at ∼0.6 eV below the ion-pair potential.^52^ Furthermore, (^2^E′) and (^2^E″) NO_3_* excited states can be accessible in coincidence with the NO ground state, ∼3.4 eV and ∼4.4 eV, respectively, below the initial ion-pair potential.^53–55^ Neutralization-induced vibrational excitation, as well as nonadiabatic dynamics, can lead to dissociation and formation of three neutral products. An electron-impact detachment study of NO_3_^–^ reported primarily intact neutral NO_3_, but also NO_2_ + O yields as well as a minor contribution of NO + O_2_ that requires structural rearrangement of the initial nitrate anion geometry.^56^ The latter channel yield was reported to increase when the electron impact energy was increased from 15 to 25 and 35 eV.^56^ Thus, NO^+^ + NO_3_^–^ MN reactions can dissociate an N–O bond, leading to three-body product channels. The three-body NO + NO_2_ + O ground-state lies 3.4 eV below the ion pair.^50,51,53^ Furthermore, electronically excited states of NO_2_, NO, O, and O_2_ may be formed,^51,57,58^ a complete table of the 15 energetically accessible product channels is provided in the SI.

Here, we report an experimental study of isolated NO^+^ and NO_3_^–^ MN reactions, implementing merged ion beams that are trapped at the double electrostatic ion-beam storage ring (DESIREE).^29^ Performing the reactions in a fast-moving beam makes it possible to detect in coincidence the neutral products of individual MN events using a time and position-sensitive detector. A detailed analysis of 3D coincidence imaging of the correlated neutral products recoil from the center of mass revealed a single predominant NO + NO_2_ + O channel in its electronic ground-state. Moreover, analysis of the three-body momentum correlations allowed us to identify the intricate nonadiabatic mechanism of this MN reaction.

## Results and Discussion

Both NO and NO_3_ radicals can be stable; nevertheless, all the measured neutral products associated with MN were found to originate from three-body events and attributed to dissociation of electronically excited NO_3_ intermediates. This, in contrast to electron-impact and photodetachment neutralization of the nitrate anion that produce a substantial yield of undissociated NO_3_ radicals.^55,56,59,60^ All of the measured signals of coincident neutral pairs were found to correspond to partially detected three-body events due to the finite detector efficiency.^35,36^

Following Bogot et al.,^36^ a principal component analysis (PCA) of the measured multidimensional data was used to disentangle potentially competing contributions from the energetically accessible product channels. However, within the statistical errors, only a single predominant three-body channel was identified. Based on center of mass (CM) momentum conservation considerations and momentum correlations, we attributed the measured three-body events to the NO + NO_2_ + O channel. The assignment is further supported by comparing the measured kinetic energy release (KER) with the expected maximal excess energy available for the different channels, e.g., the measured KER makes the formation of excited NO* and the N + O + NO_3_ channel energetically impossible. Further details about the partially detected three-body events analysis and the assignment of the three-body channel can be found in the SI. Figure 1 presents the measured distribution of KER in NO + NO_2_ + O events. The distribution peaks at ∼2.5 eV, which is below the maximal 3.4 eV of excess energy in the reaction. Assuming electronic ground state products, the KER distribution is attributed to internal vibrational excitation of the neutral molecular products with a distribution peaked at ∼0.9 eV.

**Figure 1:**
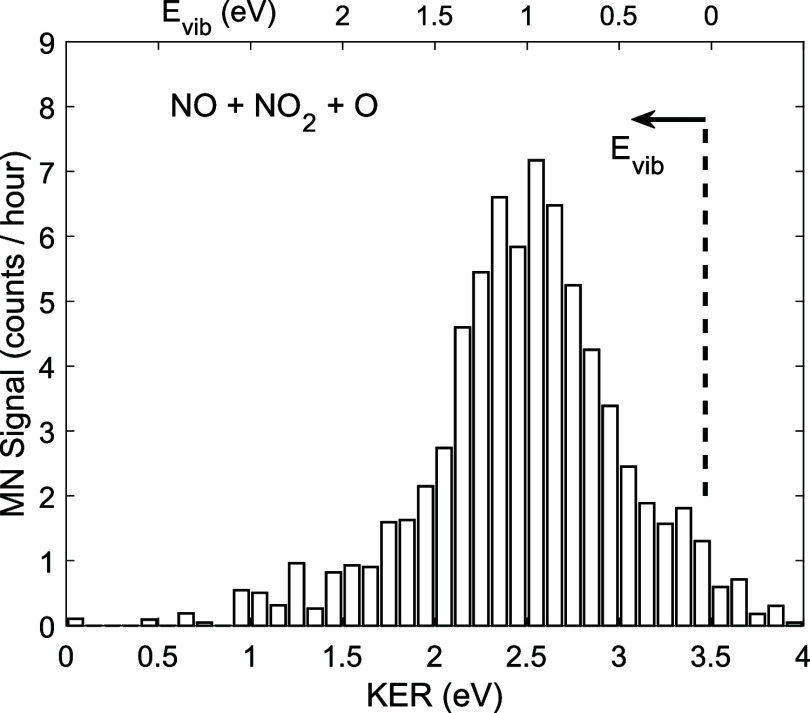
Total kinetic energy release. Background-subtracted kinetic energy release distribution of the three-body coincidence breakup events. The KER was derived from the measured 3D distances between the neutral NO + NO_2_ + O products, selecting coincidences for which the total CM momentum is conserved. The distribution is centered at ∼2.5 eV, corresponding to all products being formed in their electronic ground states and with ∼0.9 eV average vibrational excitation energy (*E*_vib_).

Further insight can be gained from analysis of how the total KER is shared among the three NO, NO_2,_ and O products. Analysis of the three-body momentum correlations using the mass-scaled Dalitz-plot representation can provide detailed mechanistic insight into the dissociation dynamics.^36,61–63^ Figure 2 shows the Dalitz-plot analysis of the coincident NO, NO_2,_ and O products. The three axes of the mass-scaled Dalitz-plot indicate the ε_NO_, ε_NO_2__, and ε_O_ kinetic energy fraction of each of the three products, scaled relative to the maximal energy allowed for each mass according to total momentum conservation.^36,64,65^ In this representation, an uncorrelated distribution of three-body dissociation events would appear as a uniform distribution within the unit circle.^36,64,65^ In contrast, the measured MN events exhibit a highly correlated distribution concentrated at the top of the vertical (ε_NO_) axis, indicating that the NO product carries nearly the maximal possible kinetic energy allowed by total momentum conservation. Moreover, the predominantly uniform distribution along the horizontal direction indicates a random sharing of the remaining energy between the NO_2_ and O, while conserving the total momentum. This distribution is characteristic of sequential dissociation mechanisms.^36,62,65,66^ Here, the sequential dissociation scenario corresponds to (1) a high KER dissociation of the NO and an intermediate NO_3_, releasing the energy gained from the attractive Coulombic potential at the ET distance; (2) a low KER dissociation of the NO_3_* intermediate, which occurs after rotational decoherence that erases any correlation between the direction of the first NO–NO_3_ dissociation and the direction of the secondary NO_2_–O dissociation. Further details about the Dalitz-plot representation and simulations reproducing the measured correlations can be found in the SI.

**Figure 2:**
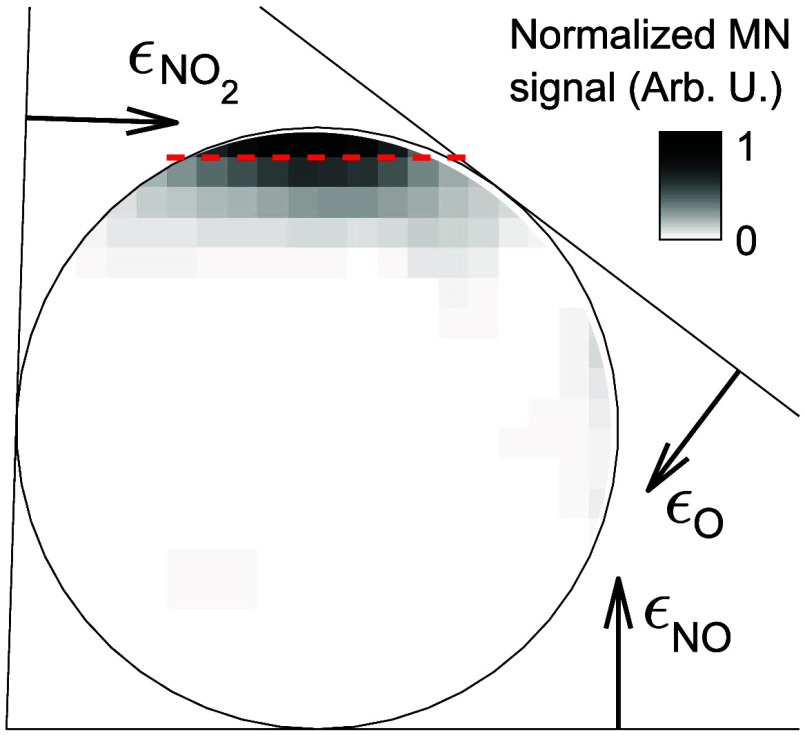
Dalitz-plot representation of the measured three-body NO + NO_2_ + O momentum correlations. Each (ε) axis represents the mass-scaled KER fraction for each neutral product, indicating the energy partitioning between the neutral products. The dashed red line represents the 90% value for ε_NO_ kinetic energy fraction.

The fact that the two dissociation stages are separate allows to extract information about the initial ET step that instantaneously stops the Coulombic attraction between the two reactants. Analysis of the ε_NO_ distribution indicates that the initial dissociation releases ∼96% of the total ∼2.5 eV KER. Assuming that the ∼2.4 eV released in the initial step reflects the energy gained from the Coulombic attraction potential, we estimated that ET occurred at a ∼6 Å distance between the ions. This is according to a purely Coulombic $-\frac{14.4\;\mathrm{eV}\cdot Å}{R\left(\mathrm{NO}-NO_{3}\right)}$ potential and neglecting the smaller effect of the relative orientation of the two ions at this distance. The black curve in the left panel of Figure 3 shows the Coulombic potential as a function of the R­(NO–NO_3_) distance. As the distance decreases, the ionic-pair potential crosses neutral-pair potentials that are indicated by the flat black, magenta, and green curves, plotted at the adiabatic difference energies between the ionic and neutral states corresponding to the NO_3_ in its ground and two relevant excited states.^50,51,53^ One can note that the high-lying NO + NO_3_ (^2^E′) potential, shown by the green curve, crosses the Coulombic curve at ∼3.4 eV and a ∼4.2 Å distance, suggesting a closer ET distance and a correspondingly higher KER in the initial dissociation step compared to the measured data. However, we should also consider the different equilibrium geometries of the ionic and neutral species, in particular, the difference in the equilibrium bond lengths of ionic NO^+^ and neutral NO is 1.06 Å and 1.15 Å, respectively.^67,68^ Assuming a vertical ET transition from the NO^+^ cation to the neutral NO in its electronic ground state, Poline et al. estimated a Franck–Condon distribution of primarily the v = 0–3 states that correspond to ∼0–1.1 eV of internal excitation.^69^ Moreover, the vibrational excitation of the neutral NO_3_ can be estimated by considering both theoretical and experimental Nitrate photodetachment spectra.^51,55,59,60^ A vertical photodetachment transition from the anion to the neutral (^2^E′) state potential leaves the neutral with up to ∼0.3 eV of internal excitation.^59,60^ While normal-mode analysis does not give reliable results for such a Jahn–Teller state manifold,^53^ Williams et al. suggest that neutralization excites the NO_3_ umbrella and symmetric stretch vibrations.^60^ Thus, as indicated by the dotted red curve in Figure 3, and in accordance with the measured 2.4 eV release in the initial dissociation, the electron is transferred near point ② in the figure, at a ∼1 eV higher potential energy and a correspondingly longer distance due to the vibrational excitation of the neutral products.

**Figure 3:**
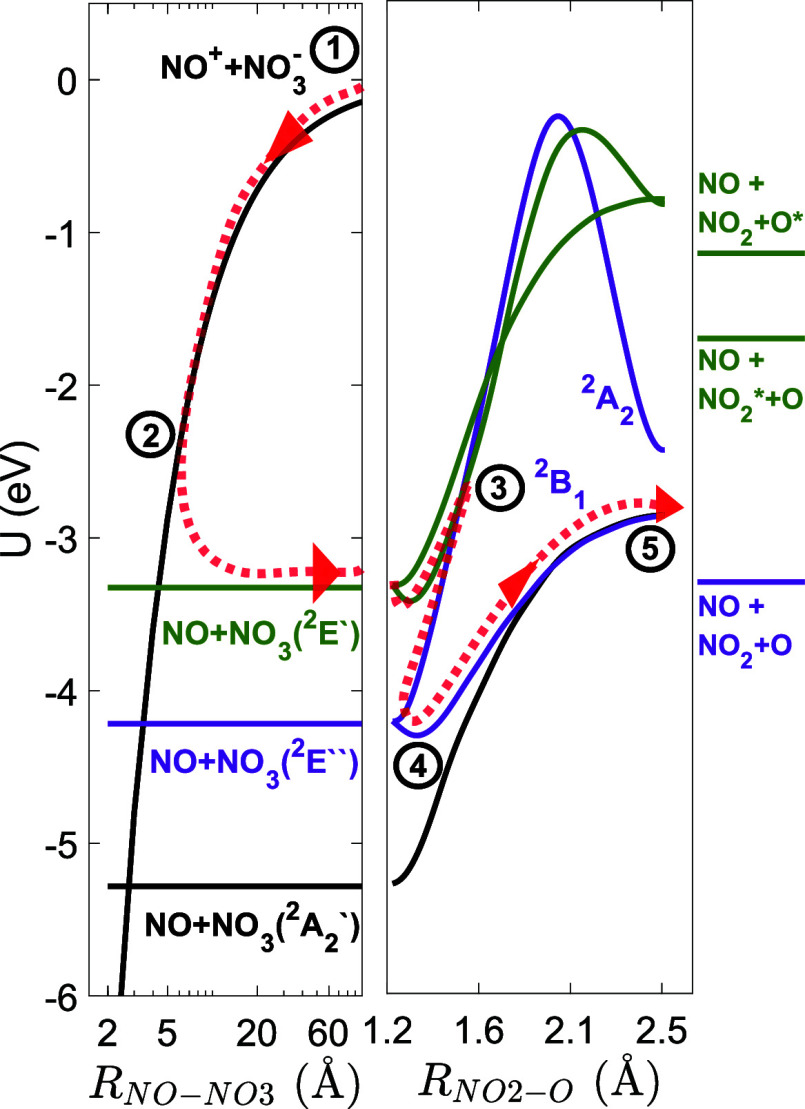
Potential energies in the MN of NO^+^ and NO_3_^–^ and suggested nonadiabatic pathway. Left panel: The black curve indicates an attractive Coulombic potential as a function of the NO–NO_3_ distance. The horizontal curves indicate the different energetically accessible electronic neutral states potentials for the neutral NO and NO_3_, neglecting dipole–dipole interactions. Right panel: Potential curves as a function of the internal NO_2_–O distance in the NO_3_ radical are reproduced from Eisfeld et al.^53^ The suggested nonadiabatic pathway which is based on the experimentally measured three-body momentum correlations is shown schematically by the dotted red curve on both panels. This pathway has five significant steps. At step ① the oppositely charged ions attract each other with a Coulombic force. Then, at a distance of ∼6 Å, marked by point ②, an electron-transfer forms a neutral ground state NO and electronically excited NO_3_ (^2^E′) both with internal vibrational excitation. Points ③–⑤ describe the suggested dissociation pathway for the NO_3_ radical. This proceeds through a nonadiabatic transition from the (^2^E′) state to ^2^A_2_ and ^2^B_1_ reduced symmetry branches which split from the (^2^E″) excited state. Following the transition, NO_3_ dissociates, yielding NO + NO_2_ + O, all in their electronic ground state.

The neutral potentials shown in the right panel of Figure 3 as a function of the NO_2_–O distance, neglecting the effect of the far-away NO product and using the ab initio multireference configuration interaction calculations of the NO_3_ system by Eisfeld and Morokuma,^53^ which reproduce the experimentally measured high-resolution photodetachment spectra.^55,60^ The ^2^E′ state is stable against dissociation to its adiabatic limits of NO_2_* + O or NO_2_ + O*.^53^ Nevertheless, NO_3_ photodissociation via the ^2^E′ state is known to be dominated by dissociation into the NO_2_ + O ground state, except for a narrow energy range in which only NO + O_2_ dissociation occurs via a roaming mechanism.^54,70,71^ Eisfeld and Morokuma predicted that by breaking the D_3h_ symmetry and elongation of one of the NO_2_–O bonds in the neutral NO_3_ molecule by 0.2 Å, near point ③ indicated in Figure 3, the system arrives at a low lying conical intersection with the ^2^A_2_ reduced symmetry potential that splits from the (^2^E″) state, shown by the purple curve which reaches the adiabatic limit of NO_2_ + O ground state.^53^ The expected ∼0.3 eV internal excitation of the intermediate NO_3_ (^2^E′) is therefore sufficient for reaching the conical intersection and producing the experimentally dominant breakup. As illustrated in the right panel of Figure 3, the direct dissociation of the ^2^A_2_ potential is prevented by a high ∼4 eV barrier. We therefore propose that the dissociation requires contraction of the elongated NO_2_–O bond back to the D_3h_ symmetry geometry (point ④ in Figure 3), from which the system can proceed on the lower lying ^2^B_1_ branch and dissociate. This indirect dissociation path, which is indicated by the dotted red curve in Figure 3, may account for a dissociation on the picosecond or longer time scale.^72^ Thus, it can account for the experimentally observed loss of correlation between the NO_2_–O dissociation and the direction of the NO product due to rotations of the intermediate NO_3_.^72^ Furthermore, we note that the NO + NO_2_ + O limit potential is ∼0.1 eV higher relative to NO + NO_3_ (^2^E′) and we therefore expect that most of the intermediate NO_3_ (^2^E′) excitation is spent on the dissociation and the NO_2_ + O which are produced with little additional KER or internal excitation. This agrees with the measured three-body momentum correlations shown in Figure 2, indicating that NO carries the maximal possible KER fraction, i.e., most KER is released in the initial NO_3_ + NO breakup. It is important to note that direct population of the ground-state or lower lying NO_3_ (^2^E″) state by electron-transfer would be expected to result in a much higher KER in the initial dissociation step and would not leave sufficient energy for the sequential NO_2_ + O dissociation. Thus, it would result in two-body breakup events, which are not observed in the experimental data.

## Summary and Discussion

Coincidence imaging of the neutral products of isolated NO^+^ and NO_3_^–^ MN revealed a single product channel. While 15 possible neutral product channels are energetically accessible, only the ground state NO + NO_2_ + O product channel was produced. Analysis of the measured KER and three-body momentum correlations provided a detailed view of the nonadiabatic reaction pathway. The MN reaction begins with ET at an ∼6 Å distance between the ions, followed by nonadiabatic dissociation of the NO_3_ (^2^E′) intermediate. Most of the energy gained from the neutralization is released in the first dissociation step, with ∼0.9 eV of product internal excitation primarily assigned to excitation of NO vibrations in the vertical ET step.

A priori, one could also consider a potential contribution of the ONO_2_^–^ peroxynitrite isomer that is separated by a high, over 4 eV, barrier from the NO_3_^–^ nitrate geometry.^73^ While present in biological environments,^74^ the isolated peroxynitrite isomer lies ∼2 eV above the ground state nitrate isomer,^73,75,76^ making it thermally less likely to form even in a hot ion source. Furthermore, the higher initial energy would be expected to result in a substantially higher KER compared with the observed MN events. We therefore conclude that peroxynitrite isomers do not contribute significantly to the observed MN reactions.^56,59,60,77^ The detailed picture of the isolated NO^+^ + NO_3_^–^ MN reaction mechanism via an ET mechanism can provide preliminary intuition also for NO^+^ +NO_3_^–^ MN in the presence of water: The observed single product channel indicates a nonstatistical mechanism. Further theoretical studies are required to explain the apparent absence of energetically accessible channels, specifically of intact NO_3_ or O_2_ products that were observed in other nitrate neutralization studies, including photodetachment or electron impact detachment.^56,59,60,77^ The reaction of the electronically excited NO_3_* intermediates with water molecules would open additional decay channels, including HONO formation, which can compete with the intricate nonadiabatic dissociation observed here for the isolated system. Furthermore, the observation of a single product channel in the isolated reaction encourages future MN experiments of increased complexity using microhydrated cations such as (H_2_O)*_n_* NO^+^ that will make it possible to explore the formation of HONO by a mechanism of proton-transfer to the nitrate anion.^39–43^

The rich nonadiabatic dynamics initiated by MN of isolated NO^+^ and NO_3_^–^ ions, as well as of their microhydrated counterparts that coexist in Earth’s ionosphere, influence the atmospheric radical budgets. Our findings demonstrate how coincidence imaging in cryogenic merged-beam facilities can disentangle nonadiabatic reaction pathways, offering new insights into the chemical reaction dynamics underlying atmospheric ion chemistry.

## Experimental Setup

The merged beams DESIREE setup has been described in detail.^29,35,36,78^ Briefly, NO^+^ ions were produced using an N_2_ and O_2_ gas mixture in an electron cyclotron resonance (ECR) ion source. NO_3_^–^ ions were formed using a N_2_O and O_2_ gas mixture in a cold cathode discharge source. The NO^+^ and NO_3_^–^ ions are accelerated to 14.4 and 27.0 keV beams, respectively, mass-selected and injected into the DESIREE rings that are shown schematically in Figure 4a. The ions that are formed in internally excited states cool within the first few hundred ms of trapping, after which the collected data can be attributed to cold molecular ions,^22–28,62^ see SI for more details. A 76 mm drift tube is supplied with a +900 V potential (*U*_0_) that accelerates the anion and decelerates the cation beams, thus reaching velocity matching conditions and sub-0.1 eV collision energies.^26,27,35^ In the remaining merged section, the collision energy is ∼20 eV, resulting in substantially suppressed MN reaction rates and strong angular preference toward forward NO and backward NO_2_/O ejection, as opposed to the expected isotropic angular distribution from the low-energy MN reactions of interest.^35,79^ Neutral MN products are detected on a 75 mm microchannel plate (MCP) detector, located 1.856 m downstream of the drift tube. The flight time to the detector was on the order of ∼6 μs; we therefore assumed that all of the dissociation on the sub-ns time scale occurred within a mm from the initial collision point. The time and position-sensitive MCP is readout by a phosphor anode and a timepix3 camera.^80,81^ Figure 4b shows a representative timepix3 event, in which the color coding indicates the time of arrival recorded at each individual position pixel. Each of the three coincidence fragments, assigned to NO + NO_2_ + O, activated multiple pixels on the camera that records the time of arrival. The use of a threshold to determine the time of arrival results in earlier detection of higher intensity pixels in the center and later times in the lower intensity rim of each hit. Furthermore, the substantial MCP gain fluctuations contribute to additional time uncertainty due to different light intensities emitted from different hits. To overcome this uncertainty that can amount to up to a few tens of ns, Figure 4c shows how fragment hit times are determined by extrapolating the measured times of all pixels assigned to each individual hit to the infinite intensity limit. Where the infinite intensity limit corresponds to the limit of vanishing inverse of the measured time above the threshold that is recorded for each pixel. Thus, similar to position centroiding that exceeds the single-pixel position resolution,^82,83^ we typically achieve a resolution that is better than the single 1.5625 ns time clock bin of the timepix3 for individual neutral product time hits, see SI for more detail. In each event, assuming a specific product channel allows mass assignment by selecting the best permutation for CM conservation. More information on the timing resolution and the systematic effects of mass miss-assignments is available in the SI. The red lines indicate the CM position calculated for this particular event, while the blue circle indicates the distribution of the measured CM positions. Due to the finite width of the CM distribution, disentangling potential contributions of multiple channels requires further analysis of the multidimensional data.^35,36^ Figure 4d shows the result of PCA that indicated a single distribution which was assigned to the NO + NO_2_ + O product channel. Bogot et al. demonstrated the resolving power of this approach that was used to disentangle competing nonadiabatic mechanisms in hydronium and hydroxide MN.^36^ More details about the PCA method implementation for disentangling competing product channels are available in the SI, including simulated data indicating that the energetically accessible product channels would be expected to appear as distinguishable distributions in the PCA.

**Figure 4:**
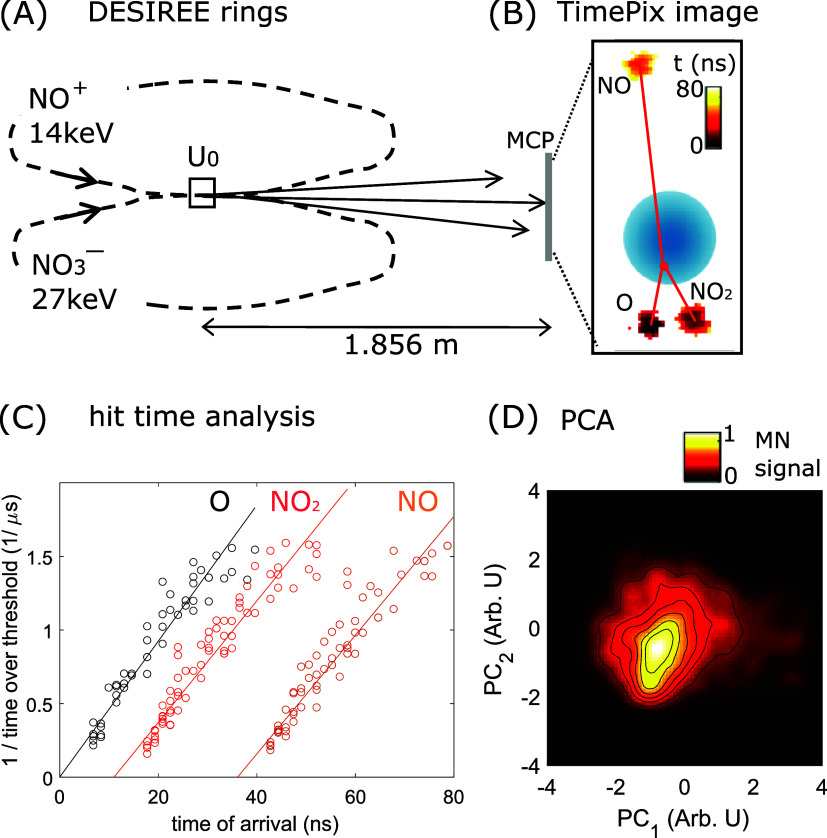
Experimental scheme. (a) Schematic representation of the merged beams setup at the DESIREE double storage ring. (b) A typical three-body NO + NO_2_ + O breakup event detected by the timepix3 camera. The blue circle shows the ion beams CM distribution, and red lines indicate the CM of the presented event. (c) Extrapolation of the measured times of arrival of individual pixels of each of the three neutral particle hits to the expected timing at the infinite intensity, or vanishing 1/time over threshold, limit. (d) PCA analysis of the multidimensional data, showing a single distribution as a function of the two most significant principal components PC_1_ and PC_2_.

## Supplementary Material



## Notes

SI includes the follwing chapters: Energetically possible product channels in the NO^+^ + NO_3_^–^ mutual neutralization; Hit time determination from Timpix time of arrival (ToA) and time over threshold (ToT) data analysis; Two-body coincidence analysis; Assignment of the three-body product channel; Three-body momentum correlations analysis. Systematic effects of mass miss-assignment; Analysis as a function of trapping time (PDF)
