# Supplementary material for: The Intricate Nonadiabatic Dynamics of NO+ and NO3 – Mutual Neutralization
Source: J Am Chem Soc. 2026 May 25;148(22):22841–8. doi: 10.1021/jacs.6c03637 (PMC13322132; doi:10.1021/jacs.6c03637)
Supplement: Supplementary file 1 [file ja6c03637_si_001.pdf]

Supplementary information for

The intricate non-adiabatic dynamics of NO^+ and NO_3^- mutual-neutralization

Alon Bogot[1], Mathias Poline[2], MingChao Ji[2], Arnaud Dochain[2,3], Paul Martini[2], Stefan Rosén[2], Henning Zettergren[2], Henning T. Schmidt[2], Richard D. Thomas[2] and Daniel Strasser[1*]

[1]Institute of Chemistry, The Hebrew University of Jerusalem; Jerusalem, 9190401, Israel.

[2]Department of Physics, Stockholm University; Stockholm, SE-10691, Sweden.

[3]Institute of Condensed Matter and Nanosciences, Universite catholique de Louvain, Louvain-la-Neuve, Belgium

*Corresponding author. Email: strasser@huji.ac.il

Contents:

1. Energetically possible product channels in the NO^+ + NO_3^- mutual neutralization
2. Hit time determination from timpix time of arrival (ToA) and time over threshold (ToT) data analysis.
3. Two-body coincidence analysis
4. Assignment of the three-body product channel
5. Three-body momentum correlations analysis
6. Systematic effects of mass miss-assignment
7. Analysis as a function of trapping time

1. The possible product channels in the NO^+ + NO_3^- mutual neutralization

Tables S1 and S2 list all the 15 energetically accessible product channels in the NO^+ + NO_3^- mutual neutralization (MN),[50-58] indicating their potential relative to the ionic NO^+ + NO_3^- ground state. Table S1 lists the two-body product NO + NO_3 channels with different energetically accessible electronic excitations of the neutral products. Table S2 lists the three-body channels that arise from dissociation of the initially formed neutral complex. These include NO_2 + NO + O, as well as 2NO + O_2 channels that require structural rearrangement, and the N + O + NO_3.

Table S1: Relative energies of the accessible two-body electronic states

NO + NO_3 channels		
#	Electronic states	Relative energy (eV)
0	NO^+ + NO_3^-	0
1	NO $(^4\Pi)$ + NO_3 $(^2A')$	-0.6
2	NO $(^2\Pi)$ + NO_3 $(^2E')$	-3.4
3	NO $(^2\Pi)$ + NO_3 $(^2E'')$	-4.4
4	NO $(^2\Pi)$ + NO_3 $(^2A')$	-5.3

Table S2: Relative energies of the accessible three-body electronic states

NO + NO_2 + O channels		
#	Electronic states	Relative energy (eV)
5	NO $(^2\Pi)$ + NO_2 $(^2A_1)$ + O (^1D)	-1.4
6	NO $(^2\Pi)$ + NO_2 $(^2A_2)$ + O (^3P)	-1.5
7	NO $(^2\Pi)$ + NO_2 $(^2B_1)$ + O (^3P)	-1.7
8	NO $(^2\Pi)$ + NO_2 $(^2B_2)$ + O (^3P)	-2.2
9	NO $(^2\Pi)$ + NO_2 $(^2A_1)$ + O (^3P)	-3.4
NO + NO + O_2 channels		
10	NO $(^2\Pi)$ + NO $(^4\Pi)$ + O_2 $(^3\Sigma_g^-)$	-0.3
11	NO $(^2\Pi)$ + NO $(^2\Pi)$ + O_2 $(^1\Sigma_u^-)$	-1.0
12	NO $(^2\Pi)$ + NO $(^2\Pi)$ + O_2 $(^1\Sigma_g^+)$	-3.5
13	NO $(^2\Pi)$ + NO $(^2\Pi)$ + O_2 $(^1\Delta_g)$	-4.1
14	NO $(^2\Pi)$ + NO $(^2\Pi)$ + O_2 $(^3\Sigma_g^-)$	-5.1
N + O + NO_3		
15	N (^4S) + O (^3P) + NO_3 $(^2A')$	-1.0

Table S3 lists the relative energies in

the peroxynitrite (ONO_2) geometry of the anion and neutral systems. As the isolated anion isomer lies ~2 eV above the nitrate ground-state, this initial state would result in a higher KER. Moreover, the relative energy of the neutral at the ONO_2 isomer geometry ground state is more stable compared with the NO_3 ground state,[73-76] also suggesting a substantially increased KER for the peroxynitrite isomer.

Table S3: Relative energies of the relevant ONO_2 isomers

Peroxynitrite channels	
Electronic states	Relative energy * (eV)
$NO^+ + ONO_2^-$	~ +2
NO ($^2\Pi$) + ONO_2	~ -10.8

* The value for ONO_2^- relative to NO_3^- is debated between 2.2 eV in reference 73, and 1.7 eV in references 75 and 76.

2. Hit time determination from timpix time of arrival (ToA) and time over threshold (ToT) data analysis.

The time of arrival (ToA) resolution of the TPX3 based detector is crucial in order to accurately analyze the 3D coincidences. As shown in the experimental setup section, each neutral fragment activates a number of pixels on the TPX3 camera. To avoid the systematic time-lag of low intensity signals given a fixed threshold, for each fragment the arrival time is determined by using an extrapolation of a ToA for an infinite time over threshold (ToT) extrapolation by a linear regression of ToA as a function of ToT. The blue bars in Figure S1 show the distribution of statistical uncertainties δt for the extrapolated fragment arrival times of the measured single hit events. The red shaded region shows distribution of hits with at least a three-pixel radius. For these events, the timing uncertainty is typically below the 1.5625 ns clock period of the TPX3.

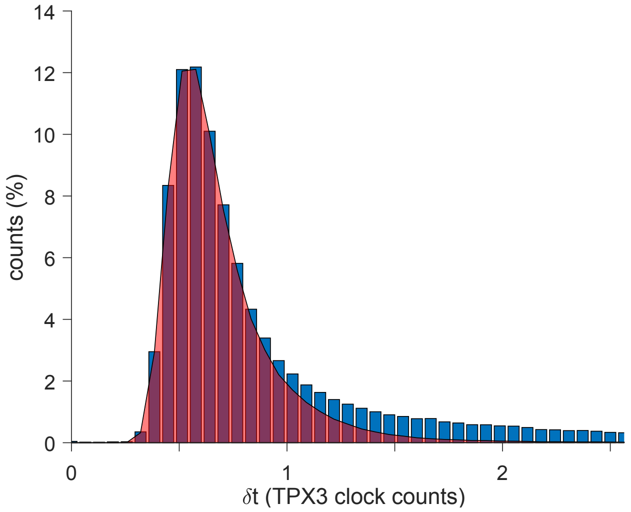

Figure S1: TPX3 statistical timing error obtained with inverse time over threshold extrapolation. *Blue bars represent the distribution of the statistical time uncertainty, calculated for individual hits. The shaded red patch shows only events which have radius of 3 pixels or above.*

3. Two-body coincidence analysis

The black curve in figure S2 shows the KER distribution obtained by analysis of the two-body coincidence events detected as stable NO + NO_3. Following Bogot et al,[34,35] the red curve shows the estimated contribution of partially detected three-body NO + NO_2 + O events to the two-body spectrum, calculated based on analysis of the measured three-body coincidences and a finite ~30% MCP detection efficiency.[34,35] Within the experimental ~0.5% uncertainty, all the measured two-body coincidences can be attributed to either random coincidence background or to partially detected three-body events. We therefore concluded that there is no substantial contribution of NO + NO_3 events.

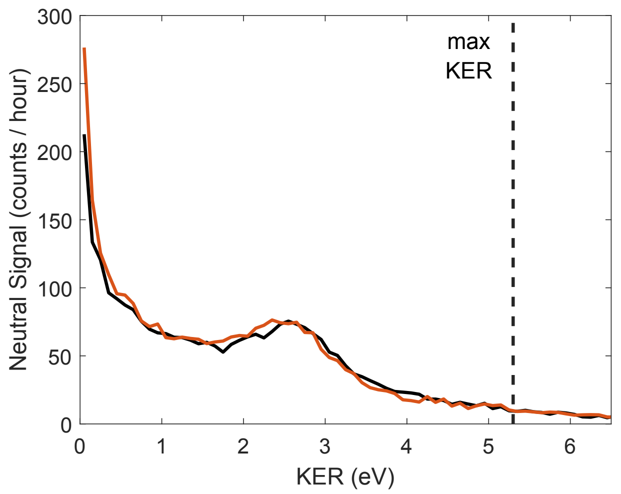

Figure S2: Kinetic energy release analysis of two-body coincidence events. *The black curve shows the measured two-body KER spectrum constructed assuming NO and NO_3 masses, while requiring CM momentum conservation. The red curve shows the estimated contribution of partially detected background calculated based on the measured three-body NO + NO_2 + O events.*

4. Assignment of the three-body product channel

A priori, the measured three-body coincidences could be assigned to either the NO + NO_2 + O, the 2NO + O_2, or the N + O + NO_3 product channels listed in table S2. The limited available excess energy in the only N + O + NO_3 channel excludes the possible contribution from this high lying channel. Figure S3 shows the KER spectrum assuming the N + O + NO_3 channel. Clearly, the KER is substantially higher than the maximal available energy in this high lying channel, indicated by the vertical line in figure S3, making it an unlikely assignment.

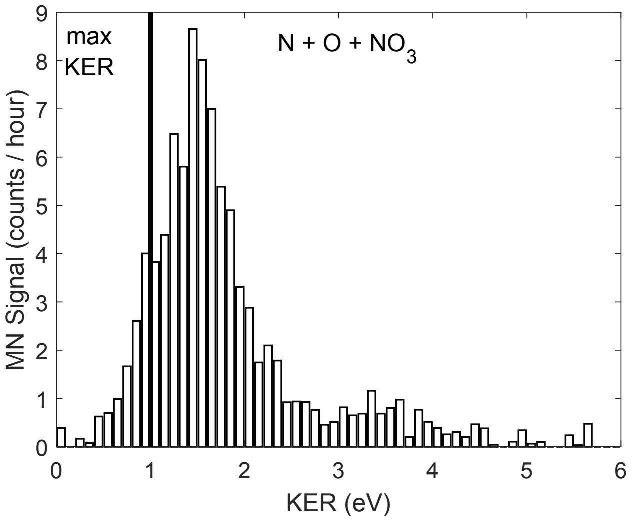

***Figure S3: Kinetic energy release analysis of three-body coincidence events N + O + NO_3 product channel**. KER spectrum under the N + O + NO_3 assignment, with most events exceeding the maximum allowed energy presented by the vertical black line.*

Following Bogot et al,[36] we perform a principal component analysis (PCA) to identify and disentangle the potential contributions of multiple product channels.

Table S4: Principal component analysis (PCA) coefficients.

	NO + NO_2 + O					NO + NO + O_2				
	KER	R_{cm}	ε_{NO}	ε_{NO2}	ε_{O}	KER	R_{cm}	ε_{NO}	ε_{NO}	ε_{O2}
PC_1	0.5982	0.2065	0.0062	0.0069	-0.0135	0.7502	0.1905	-0.0052	0.0061	-0.002
PC_2	-0.1654	0.3638	-0.0956	0.0225	0.0796	-0.1916	0.8874	-0.0085	-0.0033	0.0097
PC_3	-0.0613	0.9064	0.0804	-0.0174	-0.0685	-0.1035	-0.3899	-0.004	0.017	-0.0136
PC_4	0.2129	-0.0118	0.1107	0.2241	-0.3409	-0.1821	0.0517	0.7572	-0.2663	-0.3093
PC_5	0.1237	-0.0321	0.12	0.0738	-0.2015	-0.1159	0.0478	-0.1769	0.7326	-0.5826
PC_6	-0.3391	0.0344	-0.3106	-0.2205	0.5506	0.297	-0.0817	0.4027	0.1081	-0.4084
PC_7	-0.3019	-0.0337	0.6992	-0.5601	-0.1895	0.2182	0.1122	0.0834	-0.0356	-0.0279
PC_8	0.5868	0.0003	0.0899	-0.515	0.4159	-0.4553	-0.0032	0.0407	-0.0191	-0.012
PC_9	0	0	0.3533	0.329	0.331	0	0	0.3841	0.4989	0.5108
PC_{10}	0	0	0.4893	0.4557	0.4585	0	0	-0.2773	-0.3602	-0.3688

Table S4 lists the PCA coefficients obtained for the measured multi-dimensional data that was represented by 10 observables, including the total KER, the center of mass (CM) displacement (R_{cm}), and the kinetic energy fractions (ε_i) assigned to the individual fragments assuming either NO + NO_2 + O or NO + NO + O_2 products. The leading orthogonal principal components PC_1 and PC_2 show a strong dependence on the total KER values and R_{cm} center of mass deviations coefficients that are highlighted in table S4.

As explained in the experimental methods section of the main text, figure S4a shows the measured distribution as a function of the two leading components PC_1 and PC_2, clearly displaying a single compact feature that indicates the absence of competing processes. In comparison, figure S4b shows analysis performed on a Monte Carlo simulation considering three possible competing processes. The first process described in the main text assumes an ET producing excited NO and NO_3 intermediates and releasing 2.4 eV. The ET is followed by a sequential dissociation of NO_3 to NO_2 and O releasing an additional 0.1 eV. The second scenario begins with the same ET step, but continues to dissociation of the NO_3 intermediate to the lower lying NO and O_2 ground state, as excited O_2 ($^1\Sigma_g^+$) molecular oxygen state is unlikely as demonstrated in NO_3 photodissociation experiments.[58] The third process simulates a peroxynitrite (ONO_2) isomer, starting with an electron transfer producing NO and ONO_2 neutral intermediates. Where the ONO_2 undergoes a sequential dissociation to form the NO and O_2 products. The clearly visible three features indicate that the PCA method can be expected to distinguish between the competing processes, further supporting the PCA resolving power that was already demonstrated in earlier MN studies.[36]

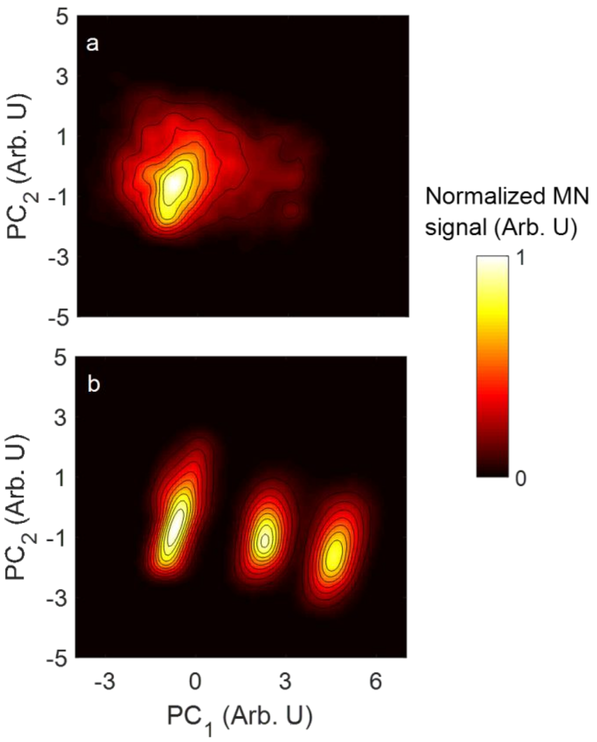

Figure S4: Principal component analysis (PCA) of three-body coincidence events. *Panel (a) shows the experimental distribution as a function of the two*

leading PCA components PC_1 and PC_2, showing a single compact feature. Panel (b) shows the same distribution resulted from a Monte Carlo simulation of three competing scenarios: (1) the proposed NO + NO_2 + O mechanism; (2) an alternative 2NO + O_2 breakup; and (3) MN with ONO_2^- yielding NO + ONO_2, followed by ONO_2 dissociation to NO + O_2.

The simulated NO + NO_2 + O channel contribution corresponds to the left most feature in figure S4b, appearing at the same position on the PC_1, PC_2 plane. To further test our assignment, we compare the likelihood of assignment by applying an increasingly stringent CM requirement. Using a loose center of mass cut with a 6 mm radius, the number of events which can be assigned with 2NO + O_2 masses is only slightly lower compared with the number of events that can be assigned with NO + NO_2 + O masses, with ~4:5 ratio. However, when we apply a strict 2 mm CM cut, the ratio falls to ~ 1:2, thus supporting the assignment of the observed single distribution of events to the NO + NO_2 + O channel.

5. Three-body momentum correlations analysis

Figure S5 presents the Dalitz plot analysis for the three-body breakup channel assigned as NO + NO_2 + O. Figure S5a shows the mapping of different three-body breakup momenta correlations on to the Dalitz plot assuming the NO + NO_2 + O product channel. For different positions on the Dalitz plot, the momentum of each of the three fragments corresponds to one of the vertices of a triangle in momentum space. All events conserving both energy and momentum lie within the indicated circle that corresponds to all the co-linear three-body dissociation geometries. Figure S5b shows the measured distribution of events on the Dalitz plot presentation, similar to figure 2 of the main text. In order to confirm the proposed MN mechanism described in the main text, figure S5c shows the Dalitz plot analysis on the Monte Carlo simulation of the NO + NO_2 + O events. Both distributions on panels b and c exhibit similar features, first, the majority of events are located at high ε_{NO} values and a uniform spread between ε_{NO_2} and ε_O, that is characteristic of a sequential mechanism.

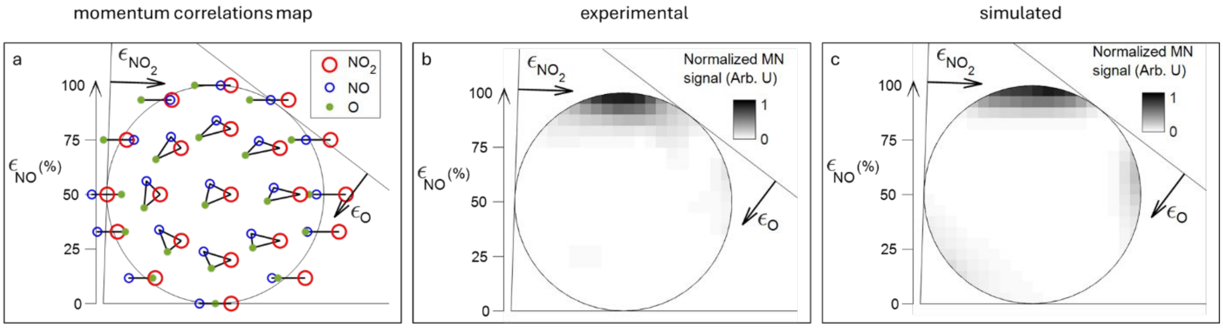

Figure S5: Dalitz plot analysis under the NO + NO_2 + O mass assignment. *Panel (a) shows the mapping of three-body breakup momenta correlations for the NO + NO_2 + O channel to different locations on the Dalitz plot. Panel (b) presents the experimental Dalitz distribution reconstructed under NO + NO_2 + O assumption, showing a concentration near the top of the NO axis and uniform sharing between NO_2 and O, consistent with a sequential breakup. Panel (c) is the Monte Carlo simulation of the proposed sequential mechanism which reproduces the experimental features, confirming the assignment to NO + NO_2 + O.*

The simulated distribution reproduces also the minor contribution of events located on the right side of the Dalitz-plot circle that correspond to high ε_{NO_2} values. Reproducing this minor feature further supports the proposed intricate mechanism. As described above, the finite width of the CM distribution limits the distinguishability of similar masses. These high ε_{NO_2} events arise due to misassigned NO_2 and NO. While it is visible in the Dalitz plot, this mass misassignment has only little effect on the measured KER. Figure S5c compares the nearly identical KER distributions obtained from the simulated events when including or excluding the potential mass-assignment error.

Three-body momentum correlation analysis allows to further evaluate the possibility of a different channel. Figure S6 shows the Dalitz plot analysis, assuming 2NO + O_2 breakup. Panel a showing the mapping of different momentum triangles to the Dalitz plot circle. Panel b shows the analysis of the measured experimental data, assuming only the 2NO + O_2 channel, while panel c shows the clearly different distribution arising from analysis of the Monte-Carlo simulated MN resulting in ground-state 2NO and O_2

channel. Here, the indistinguishable NO fragments in addition to the similar masses of NO and O_2 molecules results in difficulty to correctly assign the hits on the detector according to CM conservation considerations. Therefore, the Dalitz plot shows three distributions parallel to each of the energy fraction axes. While in the experimental measurement, we can clearly identify a large energy fraction that is carried away by one of the fragments.

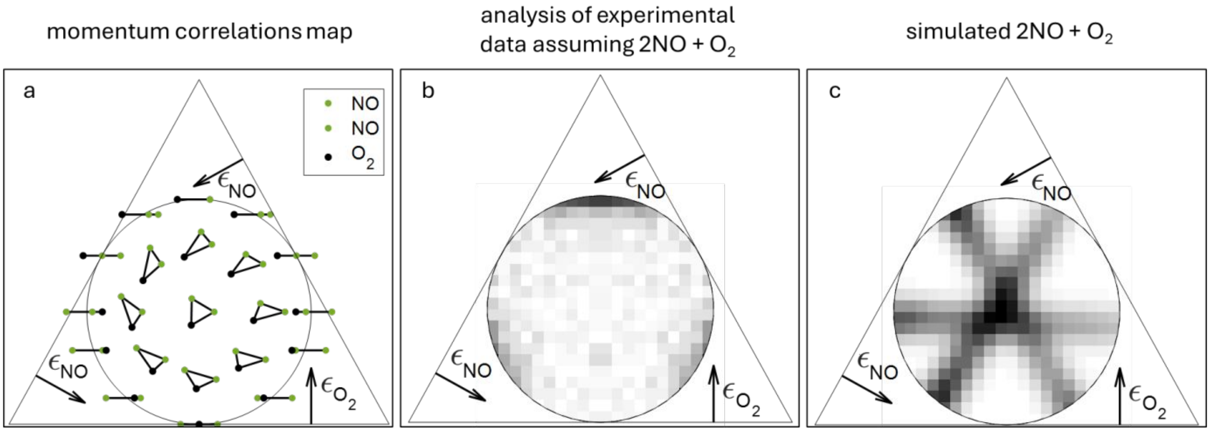

Figure S6: Excluding the 2NO + O_2 channel: Dalitz plot analysis. *Panel (a) shows the mapping of three-body breakup momenta correlations for the 2NO + O_2 channel to different locations on the Dalitz plot. Panel (b) presents the experimental Dalitz plot reconstructed under this assignment, showing that most of the KER is carried by O_2, inconsistent with the likely expected ET mechanism. Panel (c) shows the simulated Dalitz plot distribution for ground-state 2NO + O_2, substantially different from the measured data.*

6. Systematic effects of mass miss-assignment

In our experimental setup, detection of neutral fragments does not provide the mass of each fragment. Therefore, reconstruction of three-body coincidence events requires assigning fragment masses based on momentum conservation and minimization of the reconstructed CM. To evaluate the systematic effects of possible misidentifications, we examine the effect of using different mass permutations on the Dalitz plot representation. Figure S7a shows the Dalitz plot obtained using the standard analysis procedure, in which the fragment masses are assigned by selecting the permutation that minimizes the reconstructed CM displacement. This distribution is also shown in the experimental Dalitz plot in Figure 2 of the main text. Figure S7b shows the effect of exchanging the NO and O fragment assignments. This permutation typically shifts the reconstructed CM far from the detector center and therefore they do not pass the CM cut, making contribution from such miss-assignment unlikely. Figure S7c, shows the effect of NO and NO_2 exchange permutaion. Here, the resulting CM shift also leads to a reduced probability. The events that do pass exhibit a shift of the dominant population at the top of the Dalitz plot (in Figure S7a) to the right side and high ε_{NO} values. This indicates that the small population of events with high ε_{NO} values observed in panel (a) originates from occasional NO and NO_2 misidentification in the CM based mass-assignment. Finally, exchanging the NO_2 and O assignments shown in panel (d) results in only a slight reduction in the number of events and the Dalitz plot remains largely unchanged relative to panel (a). This is due to the sequential MN mechanism, where NO_2 and O fragments are typically detected close to each other due to a low kinetic energy release in the secondary NO_3 dissociation. This is also the reason that this exchange does not lead to a substantial effect on the measured KER distribution, as shown in figure S8.

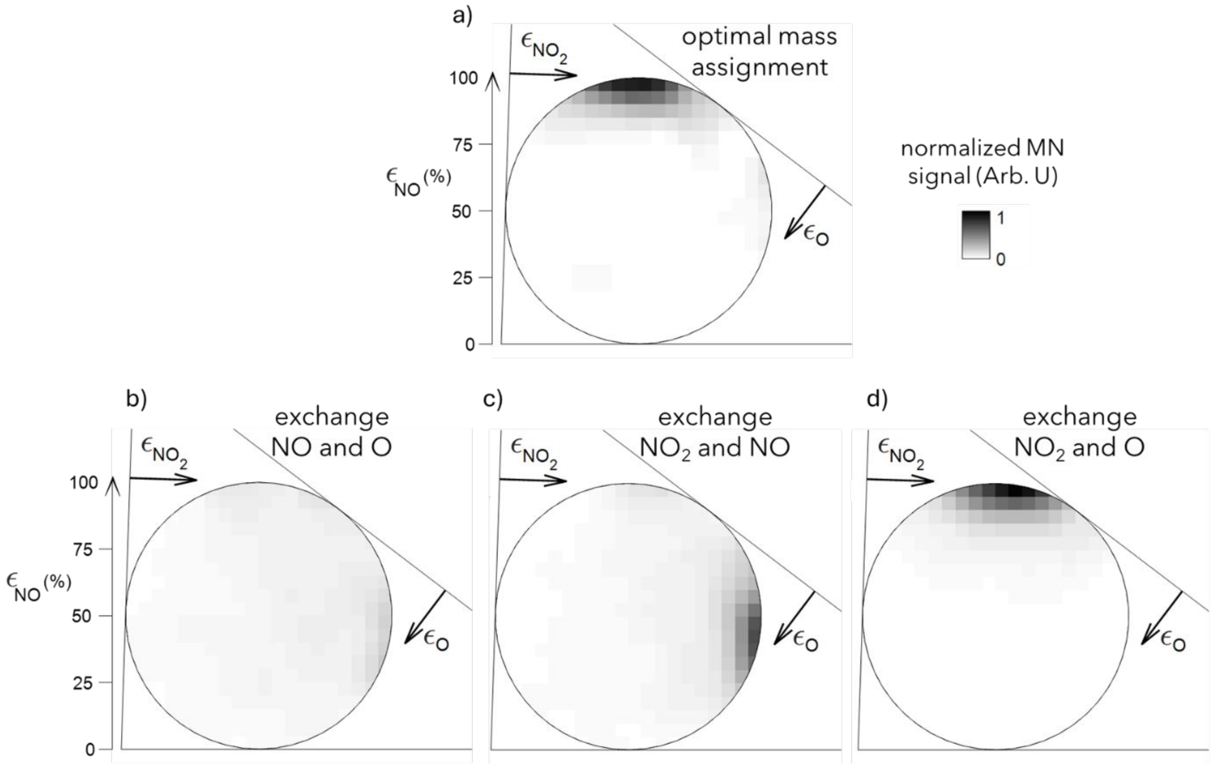

Figure S7: **mass miss-assignment effect on the Dalitz plot.** a) shows the Dalitz plot analysis with the CM based mass assignment, similar to figure 2 of the main text. b) shows the effect of NO and O exchange, c) of NO_2 and NO exchange, and d) for NO_2 and O.

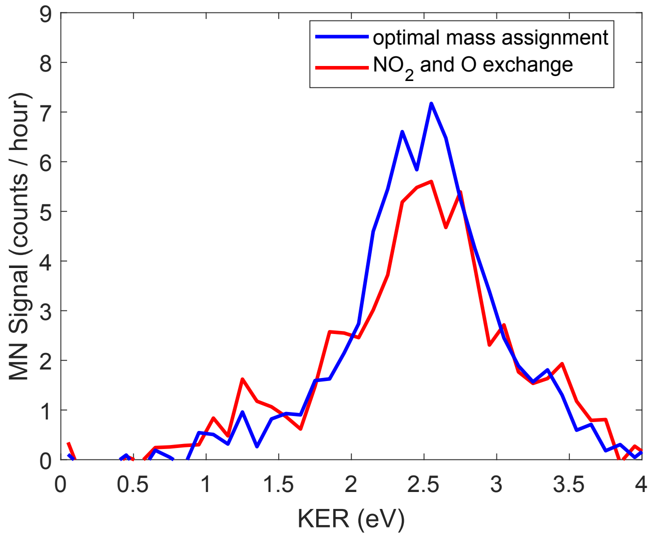

Figure S8: **NO_2 and O mass miss-assignment effect on the KER distribution.** Blue curve shows the KER distribution using the CM based mass assignment. Red curve shows the negligible effect of possible NO_2 and O exchange on the KER distribution.

7. Analysis as a function of trapping time

Figure S9 shows the time dependence of the measured neutral rate as a function of the time after the ions were injected into DESIREE storage rings. The measured data shows two distinct lifetimes. The short component, with a lifetime of $\tau_1 \approx 0.3$ s, is attributed to the decay and cooling of internally hot ions produced in the ion sources. At longer times the neutral signal is dominated by single lifetime of $\tau_2 \approx 11$ s that is attributed to the lifetime of the stored ions in the ring. To avoid potential contributions from hot ions, only data arriving after 0.5 s of storage is used for the coincidence imaging analysis. This, in a agreement with Poline et al., that attributed this time scale to the cooling of NO^+ vibrations.[34] Within the high statistical error of MN signal of hot ions in the first few hundred ms, it was not possible to identify significant deviations of the hot ion data from the reported results that are attributed to cold ions.

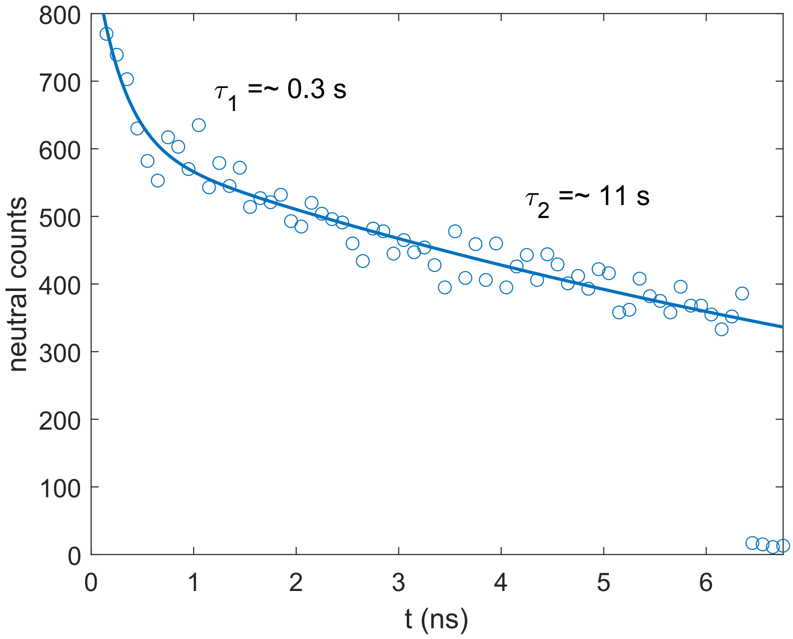

Figure S9: **Neutral yield as a function of storage time.**
